# A Standardized Protocol for the Management of Appendicitis in Children Reduces Resource Utilization

**DOI:** 10.1097/pq9.0000000000000357

**Published:** 2020-10-26

**Authors:** Christopher Pennell, Teerin Meckmongkol, L. Grier Arthur, Sean Ciullo, Rajeev Prasad, Erika Lindholm, Harsh Grewal

**Affiliations:** From the * St. Christopher’s Hospital for Children, Department of Pediatric General, Thoracic, and Minimally Invasive Pediatric Surgery, Philadelphia, Pa.; † Department of Surgery, Drexel University College of Medicine, Philadelphia, Pa.

## Abstract

Appendicitis is the most common condition requiring emergency surgery in children. We implemented a standardized protocol (SP) for treating children with appendicitis to provide more uniform care and reduce resource utilization.

**Methods:**

All patients younger than 21 years were managed with the SP beginning in January 2017. We compared data from 22 months before and after implementation. The primary outcomes included the length of stay (LOS), antibiotic days, discharge on intravenous antibiotics, utilization of peripherally inserted central catheters lines, and postoperative imaging. Secondary outcomes were protocol adherence and the rates adverse events, including postoperative abscess, return to emergency department or operating room, surgical site infection, and readmission.

**Results:**

Protocol adherence was 92.3%. For uncomplicated cases (n = 412), LOS (*P* = 0.010) and postoperative antibiotic days (*P* < 0.001) were significantly reduced. There was no difference in the rates of any adverse event (6.7% versus 2.7%; *P* = 0.058), postoperative abscess (0.4% versus 0.0%; *P* = 0.544), return to emergency department (6.3% versus 2.7%; *P* = 0.084), readmission (1.8% versus 0.5%; *P* = 0.245), or postoperative ultrasound (0.4% versus 0.5%; *P* = 0.705) and computed tomography (0.0% versus 0.5%; *P* = 0.456). For complicated cases (n = 229), the post-SP cohort had a shorter LOS (*P* = 0.015), fewer peripherally inserted central catheters lines (26.9% versus 2.7%; *P* < 0.001), fewer postoperative ultrasounds (8.4% versus 1.8%; *P* = 0.027), and fewer discharges on intravenous antibiotics (17.6% versus 0.9%; *P* < 0.001). There were no differences in adverse events before and after the SP (16.0% versus 18.3%; *P* = 0.633).

**Conclusion:**

Implementing an SP for appendicitis in children reduced resource utilization, and by inference healthcare costs, for both uncomplicated and complicated cases without adversely affecting clinical outcomes.

## INTRODUCTION

Appendicitis is the most common condition requiring emergency surgery in children, with over 56,000 pediatric appendectomies performed in the United States in 2014.^1^ The financial burden to the healthcare system is significant, with admission for appendicitis costing $6,373 for uncomplicated and $15,606 for complicated cases, respectively.^2^ Complicated appendicitis is particularly burdensome, with standard treatments historically mandating prolonged hospitalization with the administration of multiple intravenous antibiotics, resulting in significant lost time from school and work.^3^

Researchers are now challenging these standard treatments. Evidence now supports narrower spectrum antibiotics^4,5^ and early transition to oral antibiotics, with discharge based on clinical parameters rather than on predetermined therapy duration.^6^ Taken together, these departures from surgical dogma can reduce the length of hospitalization and overall resource utilization.^7^

This study’s objective was to determine whether delivering uniform and protocolized care to children with appendicitis would improve healthcare resource utilization and clinical outcomes. We hypothesized that we could achieve equivalent outcomes while using fewer resources by treating all children according to a standardized protocol (SP).

## METHODS

### Context and Subject Selection

Our institution is a free-standing children’s hospital that provides tertiary medical care to a low-income urban population that annually treats approximately 200 cases of appendicitis. We created an SP for treating appendicitis as part of a quality improvement initiative after an internal review demonstrated marked variability in management among staff surgeons. Attending pediatric surgeons, surgical fellows, physician extenders, infectious disease specialists, and pharmacists developed the protocol after reviewing the recent literature on the management of appendicitis in children. We implemented the SP in January 2017.

Patients treated from January 2015 to November 2016 made up the pre-SP cohort and were identified using International Classification of Diseases 9 and International Classification of Diseases 10 codes for acute appendicitis. Their data were extracted retrospectively from the medical record. Those treated from January 2017 to November 2018 comprised the post-SP cohort and were prospectively entered into a database following the SP’s implementation. Our local Institutional Review Board approved the study protocol (Protocol ID 1808006570).

### Intervention

**Fig. 1.**
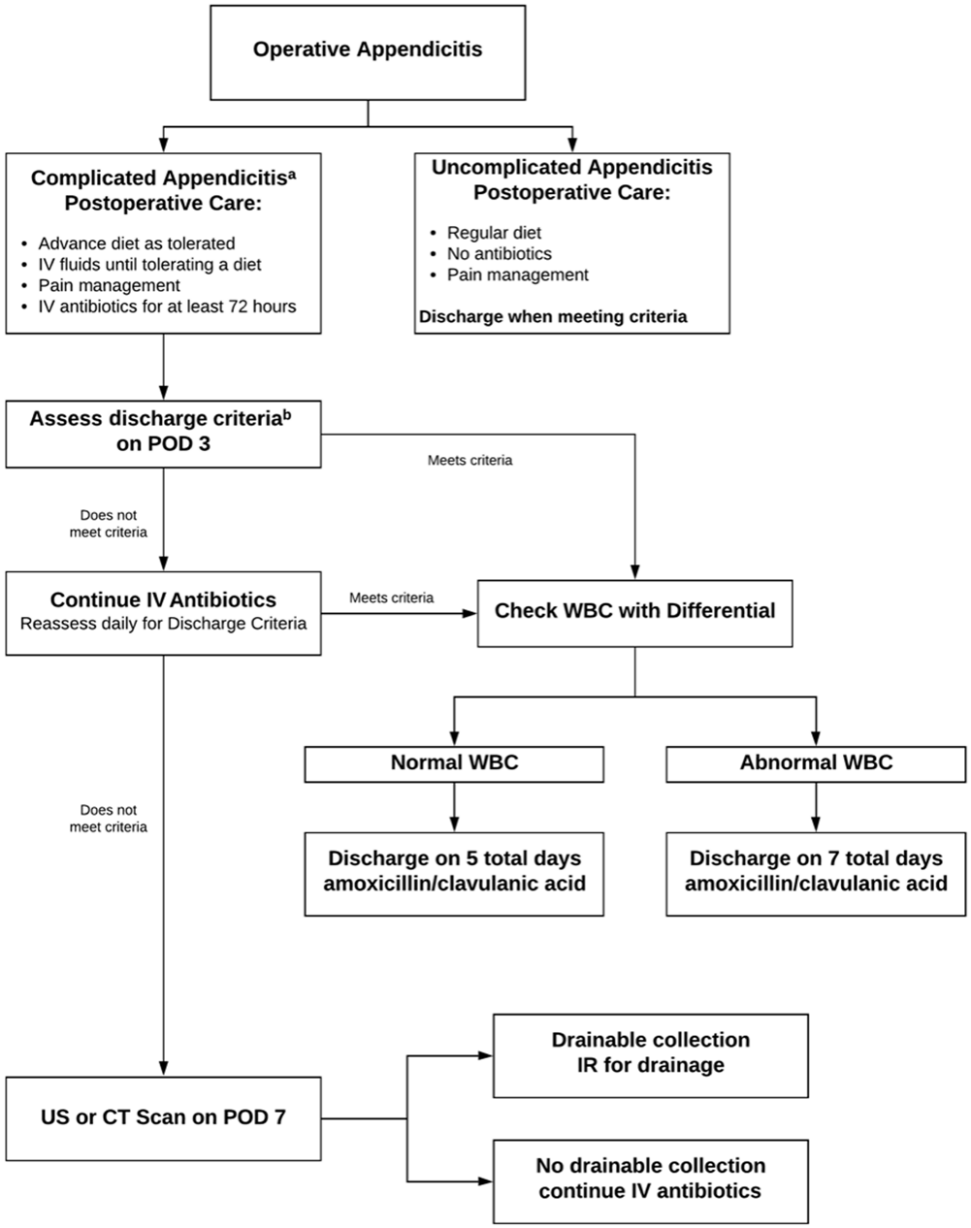
Standardized protocol for treating children with appendicitis. ^a^Complicated appendicitis includes suppurative, gangrenous, and perforated appendicitis. ^b^Discharge criteria include afebrile for 24 hours, tolerating a regular diet, pain controlled with oral medications.

The SP applies to all children treated surgically for appendicitis, including uncomplicated and complicated cases (Fig. 1). After the diagnosis of acute appendicitis, clinicians administered single preoperative doses of ceftriaxone (50 mg/kg, maximum 2,000 mg) and metronidazole (30 mg/kg, maximum 1,500 mg). They took the child for an appendectomy at the next available operating room (OR) time. For children with severe beta-lactam allergies, clinicians prescribed ciprofloxacin instead of ceftriaxone. One of 6 board-certified pediatric surgeons performed all operations. The operating surgeon classified cases as uncomplicated or complicated (suppurative, gangrenous, or perforated) appendicitis, which dictated the postoperative care. We discharged children with uncomplicated appendicitis without postoperative antibiotics once they met the prespecified criteria of being afebrile, tolerating a regular diet, and having pain controlled with oral medications.

We admitted children with complicated appendicitis to receive intravenous ceftriaxone and metronidazole for a minimum of 3 days. To be discharged, they had to be afebrile for at least 24 hours, tolerate a regular diet, and have adequate pain control with oral medications. Once they met these criteria, we discharged them on oral antibiotics (amoxicillin/clavulanic acid 45 mg/kg, maximum 875 mg), with the total duration of therapy dictated by their white blood cell (WBC) count on the day of discharge. We prescribed a full course of 5 days if the WBC count was normal or 7 days if it was abnormal. If discharge criteria were not met on post-operative day 3, intravenous (IV) antibiotics were continued, and the child was reassessed daily until post-operative day 7. We obtained an ultrasound or a computed tomography (CT) scan to evaluate for intra-abdominal abscess in children not meeting discharge criteria, despite 7 days of postoperative antibiotics. If an abscess was identified, interventional radiology was consulted for percutaneous drainage. We did not specify a specific duration of antibiotic therapy following drain placement. During the post-SP period, one of the protocol antibiotics (metronidazole) was temporarily unavailable due to a nationwide shortage. During this time, patients were treated with a modified regimen using clindamycin (10 mg/kg, maximum 900 mg) instead of metronidazole.

Early enteral feeding was encouraged with a regular diet started after surgery, and patients were allowed to autoregulate their intake. After implementing the protocol, we did not routinely place peripherally inserted central catheters (PICC) lines in patients with complicated appendicitis because situations in which one would be necessary (eg, administration of TPN, discharge on IV antibiotics) are rare. The decision to place one was left to the discretion of the attending surgeon and was generally only considered in unique circumstances.

### Study Endpoints

The primary endpoints were resource utilization measures, including the length of stay (LOS), PICC use, postoperative imaging, postoperative antibiotic days, and the proportion of children discharged on intravenous antibiotics. The secondary endpoints were the rates of adverse events, including postoperative abscess, return to the emergency department (ED) or OR, and readmission within 30 days. We defined postoperative abscess as any intra-abdominal fluid collection that required treatment with either percutaneous drainage or prolonged antibiotic therapy.

We recorded compliance with the SP, including the number of patients with protocol violations and the reasons for noncompliance. A violation occurred whenever (1) antibiotics were not administered at the correct dose, duration, or formulation (oral or IV) or (2) a WBC count or imaging study was obtained that was not indicated or was omitted when indicated by the protocol. Children treated with the modified antibiotic regimen of ceftriaxone and clindamycin were not considered protocol violations.

### Statistical Analysis

Unless otherwise noted, we report continuous data as a median with interquartile range. Continuous data were compared using Mann–Whitney U or Student’s *t* test as appropriate. Categorical data were analyzed using Fisher’s exact or Pearson’s χ^2^ tests as necessary. Results with *P* < 0.05 were considered statistically significant. Statistical analysis was performed using IBM SPSS Statistics Version 25 (International Business Machines Corp.). We displayed changes in outcomes over time using time-series analysis generated using Microsoft Excel 2010. We examined special cause variation using P charts to identify association of our improvements with implementation of the SP. We considered changes in an outcome to be highly associated with the protocol if 8 consecutive data points occurred below our preimplementation centerline in the post-SP period.

## RESULTS

### Study Cohort

We treated 699 children for appendicitis during the study period, including 57 (32 pre-SP and 25 post-SP) treated nonoperatively and therefore excluded from this report. We excluded 2 additional patients, both from the post-SP cohort. One presented in septic shock and required laparotomy with an open abdomen with multiple washouts. Another had a history of omphalocele and required laparotomy with ileocecectomy. The final study cohort had 640 patients, including 343 in the pre-SP group (224 uncomplicated and 119 complicated) and 297 in the post-SP group (188 uncomplicated and 109 complicated).

### Uncomplicated Appendicitis

Children with uncomplicated appendicitis were well-matched in the pre- and post-SP cohorts for age, WBC count, sex, race, and presence of a fecalith. Patients treated before the SP were less likely to have an ultrasound (51.8% versus 78.7%; *P* > 0.001) and more likely to have a CT scan (73.2% versus 61.2%; *P* = 0.009) preoperatively. There was no difference in the surgeon-reported operative diagnosis before and after the SP (acute appendicitis, 97.8% versus 97.3%; normal appendix, 2.2% versus 2.7%; *P* = 0.512) (Table 1). We performed laparoscopic appendectomy for all children with uncomplicated appendicitis.

**Table 1.** Demographics of Study Subjects

	Uncomplicated Appendicitis		*P*	Complicated Appendicitis		*P*
	Pre-SP (n = 224)	Post-SP (n = 188)		Pre-SP (n = 119)	Post-SP (n = 109)	
Age,* y	11.4 (8.8–15.1)	12.1 (9.7–15.4)	0.124	10.4 (7.5–14.0)	10.4 (8.0–14.2)	0.521
Admission WBC*	13.7 (9.8–16.9)	13.9 (10.3–16.8)	0.610	16.4 (13.5–19.4)	15.1 (12.1–19.8)	0.284
Male sex, %	56.7	62.8	0.211	58.0	63.3	0.412
Race, %						
White	22.8	20.2		20.2	20.2	
Black	9.8	15.4		15.1	14.7	
Asian	1.8	2.7	0.462	1.7	1.8	0.837
Other†	58.0	55.3		54.6	58.7	
Unknown	7.6	6.4		8.4	4.6	
Preoperative imaging, %						
Ultrasound	51.8	78.7	<0.001	46.2	72.5	<0.001
CT scan	73.2	61.2	0.009	74.8	65.1	0.112
Fecalith present, %	22.3	28.7	0.136	48.7	56.0	0.275
Operative diagnosis, %						
Acute	97.8	97.3		0.0	0.0	
Suppurative	0.0	0.0		22.7	15.6	
Gangrenous	0.0	0.0	0.512	12.6	19.3	0.214
Perforated	0.0	0.0		64.7	65.1	
Normal appendix	2.2	2.7		0.0	0.0	

*Values reported as medians with interquartile ranges.

†Includes mixed race patients and Latino patients not identifying as white.

Fewer children treated with the SP received postoperative antibiotics (29.0% versus 2.7%; *P* < 0.001), and LOS was significantly shorter [1.0 day, interquartile range (IQR) 1.0–2.0 versus 1.0 day, IQR 1.0–1.0; *P* = 0.010]. There were no differences in postoperative ultrasounds (0.4% versus 0.5%; *P* = 0.705) or CT scans (0.0% versus 0.5%; *P* = 0.456). No child with uncomplicated appendicitis received a PICC or was discharged home on IV antibiotics. The rate of any adverse event was not significantly different after implementing the protocol (6.7% versus 2.7%; *P* = 0.058). There were no differences in postoperative abscesses (0.4% versus 0.0%; *P* = 0.544), readmissions within 30 days (1.8% versus 0.5%; *P* = 0.245), returns to ED (6.3% versus 2.7%; *P* = 0.084), or returns to OR (0.4% versus 0.5%; *P* = 0.705) (Table 2).

**Table 2.** Resource Utilization and Clinical Outcomes

	Uncomplicated Appendicitis		*P*	Complicated Appendicitis		*P*
	Pre-SP (n = 224)	Post-SP (n = 188)		Pre-SP (n = 119)	Post-SP (n = 109)	
Length of stay,* d	1.0 (1.0–2.0)	1.0 (1.0–1.0)	0.010	5.0 (3.0–6.0)	4.0 (3.0–5.0)	0.015
PICC, %	0.0	0.0	—	26.9	1.8	<0.001
Imaging before discharge, %						
Ultrasound	0.4	0.5	0.705	8.4	1.8	0.027
CT scan	0.0	0.5	0.456	4.2	1.8	0.449
Discharge on IV antibiotics, %	0.0	0.0	—	17.6	0.9	<0.001
Postoperative antibiotic days*	0.0 (0.0–1.0)	0.0 (0.0–0.0)	<0.001	6.0 (4.0–10.0)	5.0 (5.0–7.0)	0.525
Any adverse event, %	6.7	2.7	0.058	16.0	18.3	0.633
Abscess, %	0.4	0.0	0.544	9.2	3.7	0.090
Return to ED, %	6.3	2.7	0.084	10.1	14.7	0.291
Readmission, %	1.8	0.5	0.245	3.4	3.7	0.899
Return to OR, %	0.4	0.5	0.705	0.8	0.0	0.522

*Values reported as medians with interquartile ranges.

### Complicated Appendicitis

Children with complicated appendicitis were well-matched in the pre- and post-SP cohorts for age, WBC count, sex, race, and presence of a fecalith. Patients treated before the SP were less likely to have a preoperative ultrasound (46.2% versus 72.5%; *P* < 0.001). The surgeon reported operative diagnosis was similarly distributed before and after the SP (suppurative, 22.7% versus 15.6%; gangrenous, 12.6% versus 19.3%; and perforated, 64.7% versus 65.1%; *P* = 0.214) (Table 1). Three patients (2.5%) had an open appendectomy in the pre-SP cohort, and 1 patient (0.9%) in the post-SP cohort underwent laparoscopic peritoneal washout without appendectomy due to severely complicated appendicitis. All other children underwent laparoscopic appendectomy.

**Fig. 2.**
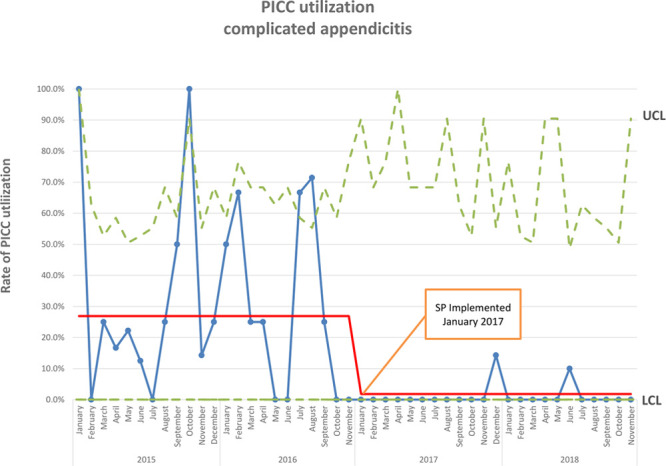
P chart of PICC use for complicated appendicitis.

**Fig. 3.**
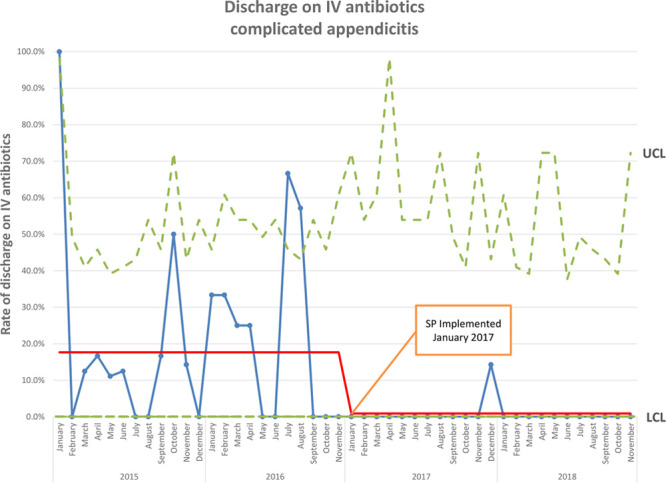
P chart of discharge on IV antibiotics for complicated appendicitis.

**Fig. 4.**
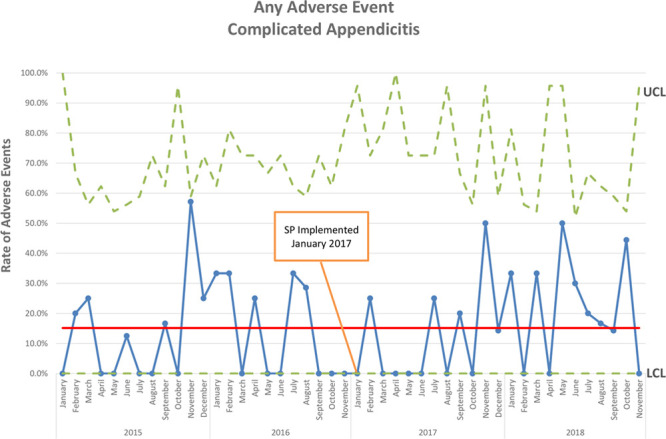
P chart of any adverse event for complicated appendicitis.

After implementing the protocol, LOS was reduced (5.0 days, IQR 3.0–6.0 versus 4.0 days, IQR 3.0–5.0; *P* = 0.015), as were the rates of postoperative ultrasound (8.4% versus 1.8%; *P* = 0.027), PICC utilization (26.9% versus 1.8%; *P* < 0.001; Fig. 2), and discharge on intravenous antibiotics (17.6% versus 0.9%; *P* < 0.001; Fig. 3) (Table 2). Only 2 children received PICCs in the post-SP cohort. One was discharged on IV antibiotics after developing a postoperative abscess. Another required a PICC for parenteral nutrition after developing an enteric fistula following laparoscopic abdominal washout with drain placement. There was no difference in the number of days of postoperative antibiotics administered (6.0 days, IQR 4.0–10.0 versus 5.0 days, IQR 5.0–7.0; *P* = 0.525) or postoperative CT scans (4.2% versus 1.8%; *P* = 0.449) (Table 2). The rate of any adverse event was not significantly different (16.0% versus 18.3%; *P* = 0.633; Fig. 4). Taken individually, no differences in the rates of return to ED (10.0% versus 14.7%, *P* = 0.291), readmission within 30 days (3.4% versus 3.7%; *P* = 0.899), or return to OR (0.8% versus 0.0%; *P* = 0.522) were identified. There was a nonsignificant trend toward reduced postoperative abscess in the post-SP cohort (9.2% versus 3.7%; *P* = 0.090) (Table 2).

### Protocol Compliance

Four surgeons completed 290 of 297 cases with an overall rate of protocol compliance of 92.3% (range 90.8%–94.3%). Two additional surgeons completed a total of 7 cases, all of which were compliant with the protocol. There were 23 protocol violations, with 17 due to antibiotic therapy. In 12 cases, these were due to incorrect antibiotic dosing or duration, in 2 cases to the wrong antibiotic formulation, and 3 cases because clinicians prescribed the wrong antibiotic. Four of the 12 children with violations of antibiotic dose or duration were receiving longer antibiotic courses that were indicated by other clinical conditions (urinary tract infection, pelvic inflammatory disease, and streptococcal pharyngitis). Two children had postoperative imaging studies not indicated by the protocol, and 4 had phlebotomy violations. We treated 22 children according to the modified antibiotic regimen of ceftriaxone and clindamycin.

## DISCUSSION

The SP for treating appendicitis presented here significantly reduced resource utilization without compromising clinical outcomes. Children with complicated appendicitis benefited the most; the SP shortened LOS by 1 day and virtually eliminated the use of PICCs, postoperative ultrasounds, and discharge on IV antibiotics. Children with uncomplicated appendicitis also benefited with a slightly reduced LOS and fewer postoperative antibiotics.

Early transition to oral antibiotics with abbreviated courses of IV antibiotics for children with complicated appendicitis is supported in the literature by a randomized trial.^8^ Several authors have shown reductions in resource utilization by incorporating this concept into comprehensive appendicitis protocols.^6,7,9^ Direct comparison is difficult due to heterogeneity in the protocols themselves, particularly with the antibiotic courses prescribed. One study discharged children on oral antibiotics once they met prespecific criteria without a protocol-mandated minimum duration of IV antibiotics.^7^ All children received 7 days of antibiotic therapy. This approach reduced LOS, PICC and parenteral nutrition utilization, phlebotomy, and postoperative CT scans. Their overall rate of adverse events was unchanged, but the intra-abdominal abscess rate decreased significantly from 24.1% to 9.8%.

In contrast, another protocol used clinical parameters to determine the need for antibiotics after discharge. Children received a minimum 3-day course of IV antibiotics and were only discharged on oral antibiotics if they had persistent leukocytosis after meeting discharge criteria.^6^ Using this protocol, the authors discharged 60% of children without antibiotics compared with 38% before the protocol. They reported no significant difference in clinical outcomes. There was a trend toward increased postoperative abscesses from 14% to 23%, raising concern for complicated appendicitis that was undertreated but not detected statistically due to insufficient power.

The protocol presented here is a hybrid of these approaches because all children receive a minimum of 3 days of IV antibiotics. However, the overall duration of their therapy was tailored based on their WBC count at discharge. The goal was to reduce the total number of antibiotic doses given and only treat children with longer courses if they had objective evidence of residual infection (eg, persistent leukocytosis) despite meeting discharge criteria. There was no difference in clinical outcomes, even though 64.2% of children received only five days of antibiotics, and only 26.7% received seven days. The remaining 9.1% received more than seven days of antibiotics. While this could be interpreted as a five-day course of antibiotics being an adequate treatment for complicated appendicitis, a higher proportion of these children had suppurative or gangrenous appendicitis which is known to have a lower risk of postoperative abscess than truly perforated cases.^10^ Therefore, treating all cases of complicated appendicitis with five days of therapy may undertreat those children at highest risk of a postoperative abscess.

Unique to this quality improvement initiative is that we reduced resource utilization for both uncomplicated and complicated appendicitis. Most protocols focus on complicated appendicitis because it is typically more morbid and resource-intensive to treat. However, most children have uncomplicated appendicitis, so reducing resource utilization in this group has the potential for significant cumulative benefit. The reductions in resource utilization for uncomplicated appendicitis reported here were statistically significant but overall quite small, raising concerns about their clinical significance. For example, the median number of postoperative antibiotic days was 0.0 for children treated before and after the protocol. The range, however, was considerably wider, resulting in a statistically significant difference. Before the protocol, 29.0% of children received at least one dose of postoperative antibiotics compared with 2.7% after the protocol. Such a reduction in antibiotic use is an essential step for antimicrobial stewardship in an era of increasing antibiotic resistance. The potential aggregate benefit is significant, given the number of children treated for uncomplicated appendicitis worldwide.

Beyond the objective improvements in resource utilization, the protocol has several less tangible benefits. As an academic institution with residents rotating continuously from multiple training programs, having a standard protocol has simplified team dynamics and communication, streamlined daily care, discharge planning, and may reduce errors. For parents, we can establish a detailed care plan and set expectations preoperatively.

This study has significant limitations that warrant discussion. As with all retrospective series, it is subject to the inherent biases resulting from retrospective data extraction for the pre-SP cohort. To minimize this bias, we collected data prospectively in the post-SP period. We also chose objective endpoints like LOS, PICC utilization, antibiotic days, and postoperative imaging to reduce bias. The lack of a specific definition for suppurative or gangrenous appendicitis is undoubtedly a potential source of bias. A standard definition for perforated appendicitis is well-established in the literature.^10^ However, there is no clear definition of suppurative and gangrenous appendicitis, making the distinction inherently subjective. Regardless of this, we treated all children with suppurative, gangrenous, or perforated appendicitis according to the complicated pathway. Thus, it is only necessary to distinguish between uncomplicated and complicated appendicitis.

Because of the nonrandomized nature of our study design, we are unable to state definitively that these changes were the direct result of implementing our protocol and not merely due to shifts in practice over time. However, the P chart shows an abrupt change in the proportion of children receiving a PICC and being discharged on IV antibiotics beginning in January 2017. This change coincided with the SP implementation and persisted through the remainder of the study period, suggesting that our protocol was responsible for these changes.

The included patients were well-matched for many demographics but did differ in their preoperative imaging. First, the lower rate of ultrasound in the pre-SP period may be due to limited access to ultrasound overnight, resulting in higher rates of CT during that time. Second, a large proportion of our patients are transferred from adult facilities after undergoing imaging, so variations in the percentage of children transferred may contribute to differences in preoperative imaging between cohorts. Notably, the large number of children transferred after a CT scan contributes to the high rate of preoperative CT compared with other published pediatric studies. Regardless, it is the operative findings not preoperative imaging that dictates postoperative care; so discrepancies in the imaging do not invalidate our study conclusions.

Finally, while the SP reduced utilization of specific health resources, quantifying the financial benefit of these reductions was not possible. Because the hospital chargemaster changed multiple times during the study period, a direct comparison of hospital charges was not feasible. Moreover, such an analysis would only include charges for inpatient care and miss the significant costs associated with administering home intravenous antibiotics in the pre-SP period.

Using the Plan, Do, Study, Act^11^ quality improvement methodology, several protocol modifications have been implemented based on the analysis of the data presented. We narrowed our definition of complicated appendicitis to include only children with a visible hole in the appendix, appendicolith in the abdomen, well-formed abscess, or diffuse pus in all 4 quadrants. Cases previously classified as suppurative or gangrenous are now treated according to the uncomplicated pathway. We made this change because no child with suppurative or gangrenous appendicitis in either cohort developed a postoperative abscess and because other authors have done this without increasing postoperative abscesses.^12^ We no longer use WBC counts to determine antibiotic duration because doing so did not significantly reduce antibiotic utilization and may have contributed to delays in discharge. We are in the process of prospectively collecting data on patients treated with this revised protocol to determine its effect on the same outcome measures reported here.

## CONCLUSIONS

We successfully implemented an SP for treating appendicitis in children. The uniform care provided by this protocol reduced resource utilization, and, by inference, costs to the healthcare system, for uncomplicated and complicated appendicitis without compromising clinical outcomes.

## DISCLOSURE

The authors have no financial interest to declare in relation to the content of this article.

## ACKNOWLEDGMENTS

The authors thank Jenah Eastep for her assistance in constructing control charts.
